# A Gene-By-Gene Approach to Bacterial Population Genomics: Whole Genome MLST of *Campylobacter*

**DOI:** 10.3390/genes3020261

**Published:** 2012-04-12

**Authors:** Samuel K. Sheppard, Keith A. Jolley, Martin C. J. Maiden

**Affiliations:** 1 Department of Zoology, The Tinbergen Building, University of Oxford, South Parks Road, Oxford OX1 3PS, UK; E-Mails: samuel.sheppard@zoo.ox.ac.uk (S.K.S.); keith.jolley@zoo.ox.ac.uk (K.A.J.); 2 Microbiology & Infection, Institute of Life Science, College of Medicine, Swansea University, Swansea SA2 8PP, UK

**Keywords:** *Campylobacter jejuni*, *Campylobacter coli*, campylobacteriosis, whole genome sequencing, next generation sequencing, genome analysis

## Abstract

Campylobacteriosis remains a major human public health problem world-wide. Genetic analyses of *Campylobacter* isolates, and particularly molecular epidemiology, have been central to the study of this disease, particularly the characterization of *Campylobacter* genotypes isolated from human infection, farm animals, and retail food. These studies have demonstrated that *Campylobacter* populations are highly structured, with distinct genotypes associated with particular wild or domestic animal sources, and that chicken meat is the most likely source of most human infection in countries such as the UK. The availability of multiple whole genome sequences from *Campylobacter* isolates presents the prospect of identifying those genes or allelic variants responsible for host-association and increased human disease risk, but the diversity of *Campylobacter* genomes present challenges for such analyses. We present a gene-by-gene approach for investigating the genetic basis of phenotypes in diverse bacteria such as *Campylobacter*, implemented with the BIGSDB software on the pubMLST.org/campylobacter website.

## 1. The *Campylobacter* Problem

Campylobacteriosis, caused by infection of humans with *Campylobacter* species, is one of the most common forms of bacterial gastroenteritis worldwide, affecting large numbers of people in both industrialized and non-industrialized countries [1]. The burden of this food-borne disease in the UK, for example, has been estimated at more than 400,000 cases annually, at a cost of some £580 million each year to the economy [2]. Although the majority of those infected experience only mild gastroenteritis, more severe forms of diarrhea also occur, as do severe systemic infections with sequelae that include flaccid paralysis [3]. Despite the importance of the disease it was not described until the mid-1970s [4], and remains poorly controlled. Consequently, reducing transmission of campylobacters to humans remains a priority for public heath, food production, and health care sectors [2]. There are ongoing attempts to trial interventions to prevent human infection, but much remains to be learned concerning the fundamental biology of how these organisms infect humans, which will be essential to establish and maintain effective knowledge-based disease control [5]. A number of features of the biology of the disease-associated members of the genus *Campylobacter* have made genome-wide analyses especially important in improving our understanding of these pathogens and, as our capacity to collect whole genome sequence data increase [6], it is likely that these approaches will continue to play an important role in reducing the burden of human campylobacteriosis.

## 2. *Campylobacter* Ecology and Population Structure

The principal causes of human campylobacteriosis, *Campylobacter jejuni* (approximately 90% of cases) and *Campylobacter coli* (around 10%), are widely distributed as apparently harmless commensal components of the microbiota of birds and mammals, both wild and domestic, and are genetically and antigenically highly diverse [5]. For many years this diversity confounded the development of the reproducible typing schemes which are the essential tool of epidemiology and disease control. In the last 10 years or so, the application of nucleotide sequence-based typing, including multi-locus sequence typing (MLST) [7] and antigen gene sequence typing [8], have provided robust and reproducible means of characterizing *Campyloacter* isolates and enabled progress in understanding their biology [9].

*Campylobacter* MLST indexes the sequence variation at seven housekeeping gene fragments, each around 400 bp in size [10]. A single scheme is used for both *C. jejuni* and *C. coli* and has enabled both inter- and intra-species diversity to be defined and separate MLST schemes have been developed for other members of the genus [11,12]. At the time of writing between 300 and 600 allelic variants for the housekeeping loci in the *C. jejuni/C. coli* scheme had been described, present in more than 5,500 combinations, which are known as sequence types (STs) (for details please see http://pubmlst.org/campylobacter/). As might be expected, the diversity of surface antigen genes is higher, with more than 1,400 alleles identified for the genes encoding the flagella (*fla*) and the major outer membrane protein (*porA*). MLST data have demonstrated: (i) that high levels of horizontal genetic exchange are a major factor in the generation of genetic and antigenic diversity in *Campylobacter* [13] and; (ii) that, despite this, *Campylobacter* populations are highly structured. In the case of *C. jejuni* the STs have been categorized into more than 40 clonal complexes [10], groups of related bacteria that share a common ancestor with each other and which often share phenotypic properties, such as host association [14,15]. Intriguingly, *C. coli* exhibits a distinct population structure to *C. jejuni* [16]. The *C. coli* isolates recovered by sampling to date are less diverse, *i.e.*, there are fewer clonal complexes, which belong to one of three clades (clades 1, 2, and 3), with clade 1 containing at least two lineages, corresponding to the ST-1150 and ST-828 complexes [17]. Such differences in population structure are intriguing in two such closely related organisms, which share about 85% nucleotide sequence identity across the genome and which apparently inhabit the same niche, the gastrointestinal tract of mammals and birds.

The application of MLST and antigen gene typing has established that certain *Campylobacter* genotypes are associated with farm animals and human disease, while other genotypes are found mainly in the environment and wild animals and are less commonly associated with human infection [10,18,19], although they do contribute to disease in rural settings [20]. In the case of *C. jejuni*, genetic attribution studies have estimated that clonal complexes associated with chickens can account for as much as 80% of human infection, probably via contaminated retail chicken meat [21,22,23]. Similarly, a large proportion of *C. coli* infection is caused by genotypes found in farm animals with isolates from the ST-828 clonal complex accounting for most *C. coli* samples from both farm animals and human disease [15]. This lineage, and the other major *C. coli* clade 1 lineage, the ST-1150 complex, has acquired substantial amounts of *C. jejuni* DNA, apparently by a process of recent genetic introgression [17]. Within food and agricultural isolates there is some evidence of host adaptation and specialization within clonal complexes that are associated with more than one host source [24].

In summary, the analysis of MLST data has provided many insights into the population structure, ecology, and evolution for *Campylobacter*, as it has for a number of other bacteria [25]. The existence of a limited number of *Campylobacter* genotypes, recognized as clonal complexes, each of which is associated with distinct phenotypes, particularly host and human disease association, provides the prospect of using association study approaches as a means of defining the genetic determinants of these interesting phenotypes. To do this, however, it is necessary to exploit whole genome data from representative isolate collections and many of the statistical tools required for this type of analysis in highly diverse bacteria are yet to be developed [26]. A major reason for this deficit is the combination of clonal descent and horizontal genetic exchange in bacterial populations, and the different role that these processes play in different bacteria [27]. Hence, conventional approaches that simply measure nucleotide changes, will overestimate the impact of genetic exchange events, potentially underestimating the impact of nucleotide changes introduced by mutation: this was why MLST used an allele based approach, simply categorizing all unique alleles with an allele number, effectively making any change, whether by mutation or recombination equivalent [28]. MLST has high power to identify members of the same lineage, which is what it was devised for, but these data have also been used to assess population genetic parameters [13,29,30]; however, the small numbers of loci do place constraints on the accuracy of this approach. While the use of model based statistical analysis approaches such as CLONALFRAME [31], STRUCTURE [32] and BAPS [33], provide methods to resolve these problems [34,35] the computational requirements of these approaches make them poorly scalable to the whole genome level, and it may be necessary to rely on allele-based rather than nucleotide sequence-based approaches for the analysis of very large numbers of whole genomes. 

## 3. Genomic Analysis of *Campylobacter* Isolates

Much of the research that has attempted to look at genome-wide patterns of variation in *Campylobacter* has, to date, involved comparative gene indexing using DNA microarrays [36,37,38,39,40,41,42]. These studies have provided evidence for genomic differences among *C. jejuni* complexes and have identified variable regions within the species which include the LOS, capsular polysaccharide, flagellar biosynthetic, and restriction-modification loci [36,37,38,39,40,41,42,43]; however, a major disadvantage of all such studies is that genes that are not present in the isolates used to construct the microarray cannot be detected. The publication of increasing amounts of sequence data has provided opportunities for further investigating genome diversity within *C. coli* and *C. jejuni*. This has provided information about the core and accessory genes [44] and revealed major structural differences that are associated with the insertion of phage- and plasmid-like genomic islands, termed *C. jejuni* integrated elements (CJIEs) [45]. To date, however, such studies have primarily focused on gene presence and absence and have not examined the rich signal of variation present in homologous yet variable sequences. 

An additional challenge is that any comparison based on a single reference isolate will divide a comparator population into two categories: those which are like the reference and those which are unlike the reference. This can lead to misinterpretations of the data, as for example, in a study in which the gene contents of 111 *C. jejuni* isolates, principally from disease and host animal sources, were compared using a microarray based on the genome sequence of the ST-21 complex disease isolate, NCTC 11168 [46]. From these data, it was argued that the population was divided into two distinct clades and that the majority of human disease isolates belonged to the ‘non-livestock clade’ rather than the ‘livestock clade’: suggesting that most *C. jejuni* infections come from non-livestock sources [36]. This finding was inconsistent with other *C. jejuni* infection research based upon risk assessment [46], outbreak investigation [47,48], analytical epidemiology [49], and attribution based upon sequence type data [20,21,22], all of which identify agricultural animals, particularly contaminated poultry meat, as the principal source of human infection. The reason why the microarray study [27] did not assign human disease to the agricultural source is that this type of analysis (*i.e.*, based on a single reference), while correctly grouping related isolates with the reference, the study categorized all less related isolates into a single additional clade’ irrespective of their wide genetic differences, a form of phylogenetic discovery bias [50]. Analysis based upon currently available DNA sequence data has shown that rather than being divided into two deep branching clades, *C. jejuni* populations are highly structured, with numerous identifiable clusters of clonally related lineages which are identified as clonal complexes by MLST. Many of these clonal complexes contain lineages from farm animals and disease which further supports the hypothesis that agriculture is the major source of disease [35]. 

A further challenge is that for such studies the choice of isolates used in the analysis is important, especially ensuring that the collections analyzed contain isolates appropriate for the analysis being undertaken. This has lead to some of the controversy over the widespread introgression of *C. jejuni* alleles into *C. coli* populations [17,44,51]. As the genetic introgression has occurred in agriculture-associated, and therefore human disease-associated, *C. coli* genotypes studies that only sample human and agricultural *C. coli* isolates have no power to detect the introgression for the following reasons. When highly similar (<4% divergent) DNA sequence is found in isolates from both species, which are approximately 12% divergent at the nucleotide level, there are two explanations: (i) that this part of the genome has not diverged and that these sequences are part of an ancestral core genome or (ii) that there has been recent introgression between the species at these loci after they diverged. By analysing more comprehensive *C. coli* isolate collections that include non-introgressed strains (≈12% divergent at all loci), it becomes clear the first explanation [35,37] is incorrect, because example strains have diverged at loci around the genome. Therefore, the regions of low sequence divergence in the introgressed strains are actually areas of recent recombination, not shared descent [17,38].

## 4. The Challenges of Analyzing Multiple Bacterial Genomes

Developments in parallel sequencing technologies provide opportunities for the genetic characterization of the whole genome of bacterial isolates [6], potentially providing the means for conducting genome-wide studies for associating genotypes with phenotypes. A number of studies with a limited number of isolates have been published [52], but multiplexed, very high throughput short-read sequencing make it possible to obtain near-complete genome sequence data for large numbers of isolates at economic cost and in an achievable time-frame. Costs for whole-genome sequencing continue to decline, and with current trends, this will soon be the most effective means of determining a seven locus MLST profile, if it is not already by the time this article is published. While complete or near complete whole genome sequences present a wide range of opportunities for improved understanding of both the epidemiology and functional biology of bacteria, there are formidable challenges in the storage and analysis of the data generated. To meet these challenges effectively there is a requirement for appropriate bioinformatics and analytical tools and databases of well-defined representative isolates, which are made available to the research community. 

## 5. Analyzing Genome Sequence Variation—The Reference Genome Approach

There are various approaches to describing the DNA sequence variation among multiple bacterial genomes. The mapping of sequence data from multiple isolates to a finished reference genome sequence enables the identification of the variable sites that differ between the reference isolate and those being compared to it relatively quickly and efficiently although, as parallel sequencing tends to be relatively error prone, this approach requires careful calling of these errors. Analyses of ‘single-nucleotide polymorphisms’ (SNPs) detected in this way have been used to compare the genomes of clinically-important pathogens, for example, a study of 6,714 ‘SNPs’ in a particular methicillin-resistant *Staphylococcus aureus* (MRSA) clone, revealed geographic structure and demonstrated the potential to trace person-to-person transmission within hospitals [53]. This approach works well: (i) when the degree of sequence variation among the genomes to be compared is low, as it is in one MRSA lineage; (ii) for monomorphic organisms e.g., *Mycobacterium tuberculosis* [54] or *Yersinia pestis* [55]; or (iii) when single lineages or clones of more diverse organisms are examined, such as the *Streptococcus pneumoniae* PMEN1 [56] clone or *E. coli* O157:H7 [57].

SNP-based mapping approaches are more problematic for the comparisons of more diverse bacteria, including the collections of *Campylobacter* isolates, which will be necessary for the investigation of the complex phenotype of host association. For example, in a recent comparison of 30 *C. jejuni* and *C. coli* genomes an estimated 250,000 ‘single’ nucleotide variants were present among the isolates (Sheppard *et al*., unpublished). Some of these will be localized within the genome as a consequence of horizontal genetic exchange whilst others will not, further complicating the analysis. That such data can be used for highly diverse pathogens has been demonstrated by a study of *Helicobacter pylori*, where this approach was applied to relate large-scale fluctuations in the *H. pylori* gene-pool to the phylogeography of the human host, but this did not extend to a detailed description of epidemiology and microevolution within the bacterium [58]. A further problem with the reference genome approach is that it relies on a finished complete genome against which variation must be mapped. The approach also suffers from the problems outlined above for microarray methods in that it cannot detect variation in those genes that are not present in the reference isolate. While this is not a problem for organisms with a ‘closed genome’, one where all isolates have essentially the same gene content such as *M. tuberculosis*, this is a major problem when analyzing diverse genotypes of bacteria with open genomes, such as *Campylobacter*, where new genes are continually found with the sequencing of additional isolates [59]. Therefore a different approach is required to describe precisely and efficiently the evolutionary relationships among the genomes of isolates of diverse organisms including *Campylobacter*. 

## 6. The ‘Reference Gene’ Approach to Genome Analysis

An alternative to the reference genome approach is a *de novo* reference-free assembly using assembly algorithms such as VELVET [60], followed by a ‘reference gene’-based analysis approach, in which the unit of comparison and analysis is the gene, rather than the genome. The word ‘gene’ can be extended here to include any identifiable sequence string, including sequences commonly found at a particular genetic locus, or given coding sequences (CDS), or other definable sequence motifs, either nucleotide or peptide. This approach catalogues and describes the variation within collections of genomes one ‘gene’, or indeed any sequence string, at a time by means of a set of reference sequences that describe known variation for that gene. This is essentially the approach used in MLST where sequence variation of fragments of genes from around the chromosome is indexed: for most MLST schemes seven such gene fragments of 400–500 bp are adequate [25]. A curated reference table for each of these gene fragments is maintained, with each new variant assigned a unique arbitrary allele number in order of description—this number therefore unambiguously identified the gene fragment as a unique defined and curated sequence string. Once defined, this particular variant is readily identified in sequence data from another isolate using easily implemented and understood algorithms such as BLAST. A further level of organization is achieved by grouping alleles into allelic profiles or STs, which describe unique, and again arbitrarily, named combinations of the alleles present at the different loci. Thus one ST designation parsimoniously describes about 3,500 bp of unique sequence data for each isolate examined, yet this sequence can be analyzed in a number of ways, including by sequence type, allelic profile, and concatenated or individual sequence strings [25]. 

Although to date mostly associated with seven-locus MLST, the gene-by-gene approach is highly scalable and can be used for any number of reference sequences up to the complete complement of a genome, in other words ‘whole genome’ or perhaps better, ‘genome-wide’ MLST: after all, multilocus does not imply a particular number of loci even though it is currently widely associated with seven locus analyses. There is no *a priori* reason to include only genes that are under stabilizing selection or present in all isolates, as in MLST, although for many analyses it is useful to group genes by function or the evolutionary forces which they experience. Extensive reference gene databases already exist for surface antigens, such as the Fla and PorA [61] antigens of *Campylobacter* [8] or the antigen and antibiotic resistance loci of the pathogenic *Neisseria* [62]. In addition, this approach can be used to index variation in both the core genome and the accessory or pan genome equally well and sets of reference sequences can be grouped into ‘schemes’ that reflect particular properties, of which MLST schemes represent just one possibility. The reference gene approach has the advantage that, as it does not rely on a single reference genome or set of genomes, genes present in some isolates but absent in others can be readily accommodated. Hence as more genome data are accumulated for organisms with ‘open genomes’ this variation is easily accommodated by the addition of entries into the catalogue. This approach also lends itself to the analysis of collections of genes grouped by any criteria, for example the ‘core genome’ could be examined in one analysis and included in a ‘core genome’ scheme, whilst those defined as ‘accessory’ could be included in a separate parallel analysis. Further, genetic variation can be analyzed across all of those genome sequences that share a particular gene or genetic element, whether or not they are closely related phylogenetically. 

The gene-by-gene approach has a number of advantages over existing methods of genome comparison that rely either on whole genome alignment and multiple pairwise comparisons [63,64], or on the identification of informative SNPs [53]. Since analysis is performed by comparing gene-length regions of the genome against the total known diversity of those regions, closely related reference genomes for mapping are not required. This allows much greater flexibility in handling and comparing genomes from diverse sources. This approach has the further advantage of being well-suited to the partial genome sequences generated by current parallel sequencing approaches. With the reference genome approach, error calling using statistical algorithms based on the relative frequencies of nucleotides at a position are necessary, as a proportion of the short reads being mapped will contain the errors inherent in current parallel sequencing technology. This is not required in the *de novo* assembly approach followed by gene-by-gene analysis, as such errors are accounted for by high-depth coverage in the assembly process, before any comparisons against reference sequences are performed. The detection of known alleles at defined loci also provides a rapid and easily-assessed validation of the data generated; in the authors’ experience, the sequence contigs that are produced are reliable and at least of comparable quality to data generated using Sanger sequencing. 

There are two main potential limitations to the gene-by-gene approach. The first is that the method will only detect variation in the loci that have been defined, so it will not yield information concerning regions of the genome outside of the coding sequences or for previously undefined genes. This is similar to mapping to a reference genome, which will also only find sequences present in the reference, but in this case can be resolved by application of gene discovery to unannotated regions which will be present in the *de novo* assembly. As the database of loci expands, so does the repertoire of genes that can be rapidly annotated. The second problem is that regions containing repeat sequences that are larger than the length of the sequence reads, are not assembled. This can result in the finished assembly containing multiple contigs with relatively short lengths. This latter issue is a technological one that will be resolved by the development of improved chemistries and protocols leading to longer read lengths. At a practical level, it is also not a major problem with *Campylobacter* which has low numbers of repeat regions, such that recent Illumina assemblies for this organism now contain as few as twenty contigs with the largest being over 500,000 bp in length. With such assemblies the large majority of coding sequences are fully contained within a contig and available for analysis. Where a coding sequence is located at the end of one of these contigs BIGSDB will mark this so that the region is available for phylogenetic analysis but an allele number will not be assigned. Finally, since the gene-by-gene approach uses the BLAST algorithm to identify sequence regions there can be issues when genes are duplicated within the genome or if two loci share an allele pool: BLAST alone will not differentiate these regions, but it is possible to define loci within BIGSDB based also on regions of sequence upstream and downstream of the coding sequence. An *in silico* PCR reaction can be defined for a locus and only regions of the genome predicted to be amplified by such a reaction will be assigned to a specific locus. 

## 7. Implementation of the Reference Gene Approach

**Figure 1:**
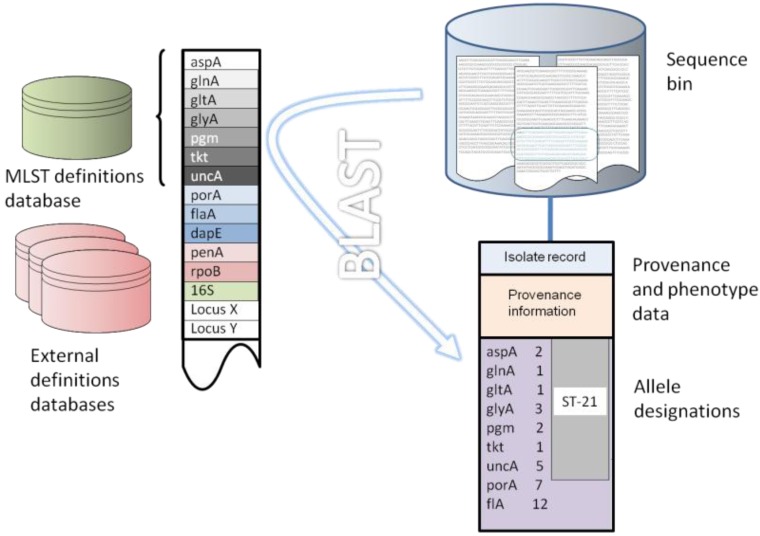
Gene-by-gene analysis of *Campylobacter* genomes. (**A**) Defined loci are entered into the database, from the finished NCTC 11168 genome; (**B**) Whole genome sequence data, such as contigs generated from parallel sequencing technologies or complete assembled genomes, are entered with isolate provenance and phenotype information; (**C**) Sequences are compared to defined loci using BLAST and allelic variants are tagged. The result is an allelic profile that catalogues variation across the genome.

There are three elements to the analysis of whole genome data using the gene-by-gene approach: (i) a repository for sequence data; (ii) an isolate record that contains the provenance and phenotypic data for each isolate for which sequence information is stored, with each sequence repository linked to an isolate record; and (iii) reference tables of predefined allele sequences for the loci of interest. This fundamental structure is implemented in the Bacterial Isolate Genome Sequence Database (BIGSDB) platform [65], which also includes the ability to link these data to other data sources such as PubMed or laboratory data. The database stores not only sequence and provenance information but also an ever-expanding set of reference loci against which each newly deposited sequence can be rapidly interrogated with computationally efficient search algorithms such as BLAST [66] (Figure 1). This structure is capable of great flexibility and expansion, limited only by the computer resources available, and its computing requirements are modest. As none of the processes are computationally intensive, and sequences are stored as strings, very large numbers of isolate sequences can be rapidly and efficiently stored and interpreted. 

The sequence repository can contain any amount of sequence data ranging from a single sequence, through multiple contigs generated from high-throughput parallel sequencing methods, to a complete finished genome. Using BLAST, sequence variants are rapidly identified and their positions within the sequence repository determined and tagged for future reference (Figure 1). Novel sequences not in the reference databases are immediately identified and can be curated and added as required. In addition to typing bacteria and identifying variation at loci of interest, the method can be used for population scale genome annotation by identifying genes and their variants, which can be grouped into schemes to reflect their function. The identification and labeling of sequences is highly scalable, since analysis time increases linearly with increasing numbers of genomes or loci and reanalysis of existing allele designations is not required as further data are added. Furthermore, since the unit of analysis is usually a single sequence, genomic data can be analyzed irrespective of the size of the assembled contigs, provided the locus of interest is fully, or mostly, contained within a single contig. This makes the approach particularly suitable for use with the current generation of parallel sequencing technologies which have relatively short read lengths that can result in genome assemblies comprising multiple contigs. Finally, since allele identification is performed by comparison of a single gene from an isolate against the entire known diversity of that locus, the method can be used to analyze highly divergent isolates, for example those from different species within the same genus. 

**Figure 2:**
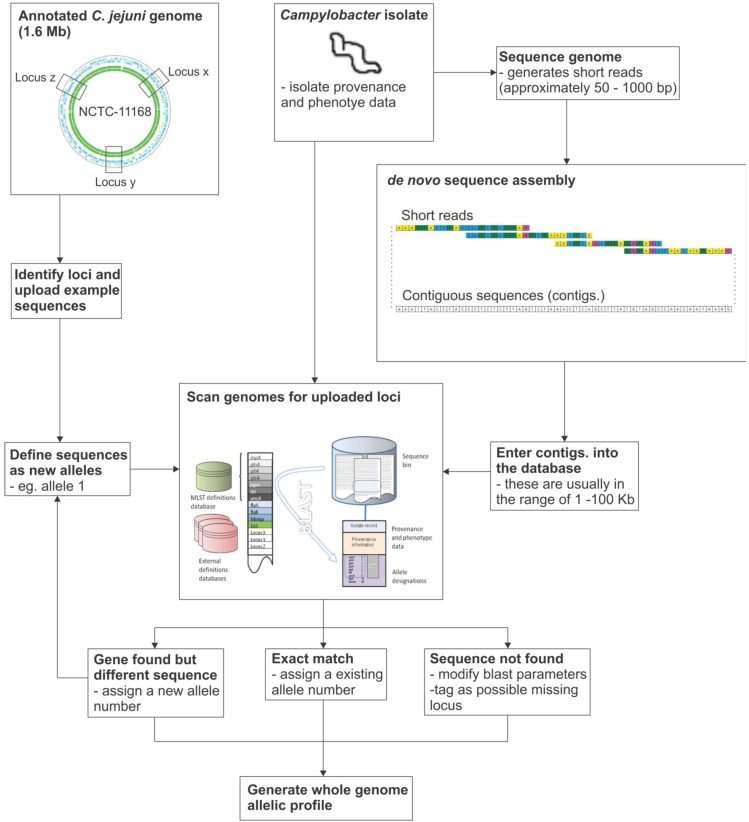
Schematic diagram of the gene-by-gene analysis pipeline.

Databases employing BIGSDB therefore replace and extend the functionality of the MLST databases that have been successfully used for over 10 years [25], as they maintain tables of curated allele sequences (nucleotide or peptide) that both catalog known sequence diversity but also act as a reference to identify the sequences present in specific isolates; however, in the case of BIGSDB, whole genome data can be stored and any number of loci can be included and placed into any number of schemes. These schemes can, for example, comprise particular sets of core genes, or genes encoding particular phenotypic properties such as biosynthetic pathways, or those encoding antibiotic resistance (Figure 2). This functionality is accessible via a web interface and the data can be linked to external data sources such as PubMed facilitating analysis of published datasets as coherent collections. The platform has been designed to handle population-scale genomic data with advanced querying of provenance and phenotype, whereas other genome-based database platforms focus mainly on characteristics of exemplar sequences from single or a few isolates of multiple bacterial species, and contain few isolates of any particular single species [67,68,69,70]. BIGSDB incorporates a number of data summary analysis and export tools which allow it to be used as a workbench for genome analysis at the population level.

The PubMLST database has been accumulating information on the population variation of *C. jejuni* and *C. coli* for a decade and continues to expand. This data archive has now been extended with the implementation of BIGSDB to include whole genome sequence information of an increasing number of *Campylobacter* isolates (more than 400 at the time of writing and expanding at a rapid rate) alongside MLST data from more than 15,000 isolates from diverse sources including clinical samples and those from animal feces and retail food products. 

The annotation of the NCTC 11168 *C. jejuni* genome [71] (1,641,481 bp) predicted 1,654 proteins. Using this information it has been possible to describe 1654 loci and assign allele numbers to all the coding sequences that can be identified in other draft or finished genomes. Work is in progress to generate reference gene sets for each of these loci and to establish a reference set of loci constituting the core genome, those genes found in the great majority of isolates. Gene discovery methods will enable the identification of those genes that contribute to the pan genome–a process that is likely to continue for some time, if it will ever be complete. Once this has been done, it will be possible to identify the distribution of sequence variants within the core genome and the presence and absence and sequence variation of accessory genes in groups of isolates associated with particular phenotypes. This gene-by-gene approach has already contributed to our understanding of the epidemiology and evolution of clinically important members of the genus *Campylobacter* and, by expanding the understanding of population genetic structure within the genus and investigating genetic variation across the genome, it will be possible to identify how phenotypic properties, such as host niche, are reflected in the population structure of these bacteria (Figure 2).

Because of the flexibility of reference gene analysis, the population genomics approach can be further enhanced by analyzing functionally related groups of genes, such as those genes involved in metabolism of a particular substrate, with fucose metabolism providing an example [72]. It is now possible to generate hypotheses about the nature of the adaptive forces that provide competitive advantages in particular host niches and to test these by examining sequence variation in metabolic genes. Continued investigation of the genomes of representative isolates shall enhance our understanding of the relationship of epidemiological phenotype to genotype for these and other important pathogens, further contributing to the control of the diseases which they cause.

## 8. Concluding Remarks

Reference-free assembly followed by the gene-by-gene analysis approach described here offers a scalable, practicable, and easily understood method for the comparison and analysis of multiple bacterial genomes that can be implemented with minimal computational resources. This approach offers an alternative to the SNP-mapping approaches, which are dependent on reference genomes and which are in any case unsuited to highly diverse bacteria such as *C. jejuni* and *C. coli*. Once assembled and deposited in a web-accessible database such as BIGSDB, genome sequence data are readily available to the community. Unassembled data can still be made available via short-read archives, but for the majority of users of these data, scientists and clinicians alike, it is assembled data that are the most useful and accessible. 

For reference sequences, once a locus has been defined, a record of the variants found at that locus can be readily maintained, as has been done for more than a decade for MLST and antigen loci. These reference sequences can be rapidly detected in any whole genome data set which they are used to query with generic and rapid search algorithms such as BLAST. As the curation process continues, more and more variants at more and more loci will be identified and defined and will act as a means of simultaneously characterizing and annotating new genome data, even when it is incomplete. Unlike SNP calling algorithms, such analyses are additive, *i.e.*, it is not necessary to re-run an analysis on the whole dataset every time a new genome is added: it is sufficient to simply query a new genome against the existing reference data set. Novel variants can be curated and added to the reference data set as part of this process, so that the reference sets continually expand as novel variants or sequence strings are identified. Importantly, this approach is an extension of existing sequence typing methods, so legacy data from exiting DNA sequence typing database such as PubMLST can be readily interpreted through a single database. The gene-by-gene approach therefore assimilates DNA sequence data collected over the last decade as well as providing a means for analyzing whole genome data and provides a practical approach to molecular epidemiological, evolutionary and functional studies in the post genomic era.
